# Meta-analysis of the strength of exploratory suicide prediction models; from clinicians to computers

**DOI:** 10.1192/bjo.2020.162

**Published:** 2021-01-07

**Authors:** Michelle Corke, Katherine Mullin, Helena Angel-Scott, Shelley Xia, Matthew Large

**Affiliations:** School of Psychiatry, University of New South Wales, Australia; South Eastern Sydney Local Health District and School of Medicine, University of Notre Dame, Australia; South Eastern Sydney Local Health District, Australia; South Eastern Sydney Local Health District, Australia; South Eastern Sydney Local Health District, Australia; and School of Medicine, University of Notre Dame, Australia

**Keywords:** Suicide, risk assessment, self-harm, suicide attempt

## Abstract

**Background:**

Suicide prediction models have been formulated in a variety of ways and are heterogeneous in the strength of their predictions. Machine learning has been a proposed as a way of improving suicide predictions by incorporating more suicide risk factors.

**Aims:**

To determine whether machine learning and the number of suicide risk factors included in suicide prediction models are associated with the strength of the resulting predictions.

**Method:**

Random-effect meta-analysis of exploratory suicide prediction models constructed by combining two or more suicide risk factors or using clinical judgement (Prospero Registration CRD42017059665). Studies were located by searching for papers indexed in PubMed before 15 August 2020 with the term suicid* in the title.

**Results:**

In total, 86 papers reported 102 suicide prediction models and included 20 210 411 people and 106 902 suicides. The pooled odds ratio was 7.7 (95% CI 6.7–8.8) with high between-study heterogeneity (*I*^2^ = 99.5). Machine learning was associated with a non-significantly higher odds ratio of 11.6 (95% CI 6.0–22.3) and clinical judgement with a non-significantly lower odds ratio of 4.7 (95% CI 2.1–10.9). Models including a larger number of suicide risk factors had a higher odds ratio when machine-learning studies were included (*P* = 0.02). Among non-machine-learning studies, suicide prediction models including fewer risk factors performed just as well as those including more risk factors.

**Conclusions:**

Machine learning might have the potential to improve the performance of suicide prediction models by increasing the number of included suicide risk factors but its superiority over other methods is unproven.

## Background

In the past 5 years a number of high-quality meta-analyses have examined the statistical strength of various types of suicide prediction. Franklin et al^1^ and Ribeiro et al^2^ conducted meta-analytic reviews of longitudinal studies that reported the predictive strength of a broad spectrum of suicide risk factors including suicidal ideation and suicidal behavior. After reviewing 50 years of research they concluded that even the most well-established suicide risk factors ‘only provide a marginal improvement in diagnostic accuracy above chance’.^2^ Other authors have used meta-analysis to examine the predictive strength of validated suicide risk scales concluding that ‘the scales lack sufficient evidence to support their use’,^3^ ‘are not clinically useful’,^4^ and ‘do not fulfil requirements for diagnostic accuracy’.^5^ More recently Belsher et al synthesised 17 suicide prediction models that were developed using both training (exploratory) and testing (validation) stages and concluded that they ‘produce accurate overall classification models, but their accuracy of predicting a future suicide event is near zero’.^6^

A 2016 meta-analysis of longitudinal studies examined the predictive properties of 29 exploratory studies that retrospectively fitted two or more non-demographic suicide risk factors to suicide mortality and 24 validation studies of suicide risk scales.^7^ Although the modest pooled odds ratio (OR) of 4.8 across the exploratory and validation studies was consistent with the disappointing results of later meta-analyses,^3–6^ the authors emphasised the extent of the between-study heterogeneity in ORs and tested possible moderators including the year of publication and different study methods. They hypothesised that prediction models that included more suicide risk factors would have better predictive strength^7^ but instead found that that the number of included suicide risk factors did not explain between-study heterogeneity in ORs.^8^ However, the inclusion of both exploratory and validation studies might have obfuscated this anticipated association given that exploratory and validation methods have quite different ways of determining the number of included risk suicide factors. An unrelated limitation of the 2016 study was a lack of studies examining predictions made using clinical judgement or machine learning.^7^

## Potential role for machine learning

Machine learning is a subset of artificial intelligence research employing computer algorithms that improve automatically through experience. Machine leaning has been touted as a method to revolutionise and personalise medicine across the spectrum of healthcare.^9^ Given the difficulty in translating the large volume of research about suicide risk factors into clinically useful suicide prediction models, there is hope that machine learning may provide a solution to the intractable problem of suicide prediction.^10^

## Aims

In this meta-analysis we expand research into suicide prediction models by examining a large and representative sample of exploratory suicide prediction models derived from cohort and controlled studies conducted in population, primary care and specialist care settings. We examined the effect size, expressed as the OR of the highest risk and lower-risk groups, according to the different types of suicide prediction models (including experimental scales, machine learning and clinical judgement) and the number of suicide risk factors included in the prediction models. We also examined other possible moderators of the ORs according to different diagnostic groups, different study settings and measures of study reporting strength. Finally, we identified the suicide risk factors that were most commonly included in the suicide prediction models. In accordance with the unanswered questions posed by the 2016 meta-analysis^7^ we hypothesised that the use of machine learning and a larger number of included suicide risk factors would both be associated with statically stronger suicide predictions.

## Method

We conducted a registered meta-analysis (PROSPERO; CRD42017059665) according to Preferred Reporting Items for Systematic Reviews and Meta-Analyses (PRISMA) and Meta-analysis Of Observational Studies in Epidemiology (MOOSE) guidelines.^11,12^

### Search strategy

**Fig. 1:**
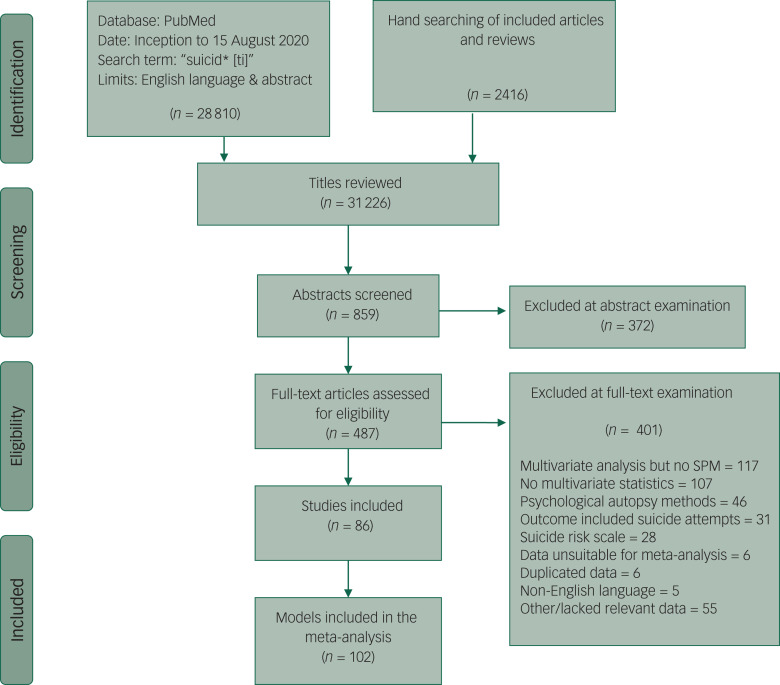
Flow chart of searches for studies reporting exploratory suicide prediction models (SPM).

Preliminary searches of Medline, Embase and PsycINFO from inception to January 2019 using the word ‘suicide’ resulted in an impractical number of hits,^13^ while searches with limits using the terms ‘prediction’, ‘model’, or ‘stratification’, missed papers that were identified in earlier reviews.^6,7,14^ In contrast, searches of PubMed using variants of the word ‘suicide’ in the title reliably located papers identified in searches of multiple databases. Therefore, in order obtain a large and representative sample of relevant studies, English language papers with accompanying abstracts listed in PubMed from inception to 15 August 2020 were identified using title searches with the term suicid* (expanded to ‘suicida[ti] OR suicidaire[ti] OR suicidaires[ti] OR suicidal[ti] OR suicidal/death[ti] OR suicidality[ti] OR suicidally[ti] OR suicidarse[ti] OR suicide[ti] OR suicide/attempted[ti] OR suicide/self[ti] OR suicide/suicide[ti] OR suicide’[ti] OR suicide's[ti] OR suicidedagger[ti] OR suiciders[ti] OR suicides[ti] OR suicides’[ti] OR suicidiality[ti] OR suicidical[ti] OR suicidio[ti] OR suicidios[ti] OR suicidogenic[ti] OR suicidology[ti]’) (Fig. 1). Two authors (M.C. and M.L.) screened full-text publications for inclusion and exclusion criteria. Electronic searches were supplemented by hand searches of the reference lists of included studies and earlier relevant meta-analyses.^1,2,6,7^

### Inclusion and exclusion criteria

We included data from studies that:

- examined groups of people who died by suicide or survived;
- used cohort or case–control methods;
- reported on a type of exploratory suicide prediction model using two or more clinical suicide risk factors (other than age and gender) or clinical judgement (assuming that clinicians consider more than one risk factor in making a clinical suicide risk assessment); and
- reported sufficient data to calculate the OR (i.e. the number of higher- and lower-risk patients that survived or died by suicide, the sensitivity and specificity, or other effect-size data).

We excluded data from studies that:

- examined validated existing suicide risk scales (including validation stages of machine-learning studies);
- reported on suicide attempts or a combined outcome of suicide and suicide attempts;
- used psychological autopsy methods;
- reported a suicide prediction model based on a single suicide risk factor combined with age and/or gender;
- only examined biological markers; or
- reported insufficient effect-size data to include in the meta-analysis.

Validation studies of suicide risk scales were excluded because the number and nature of items in suicide risk scales are not empirically derived from the validation data-set.

### Data extraction

Two authors (M.L. and M.C.) independently extracted the effect-size data that was then reconciled by a third author (S.X.). Differences in effect-size data were resolved further by examination and consensus by M.L. and M.C. Where possible the effect-size data recorded was in the form of counts of true positives and total positives, and false negatives and total negatives to allow a meta-analysis of sensitivity and specificity of the suicide prediction models. Most studies dichotomised the patients into higher and lower-risk groups but, when three or more risk strata were reported, the cut-off point associated with the highest OR was used. Two authors (M.L. and H.A.-S.) independently extracted the moderator data that was then reconciled by a third author (K.M.), with differences resolved by further examination by M.L., M.C. and K.M. and consensus. The list of suicide risk factors used in each suicide prediction model was recorded by M.L. and crosschecked by K.M. Suicide risk factors that were included in more than five suicide prediction models were considered to be replicated independent predictors of suicide.

### Moderator variables

We classified the type of the suicide prediction models according to:

- clinical judgement (including both heuristic assessments of suicide risk recorded in medical records and risk categorisations by researchers who were masked to suicide outcomes);
- multi-item experimental scales with items selected after bivariate testing of suicide risk factors;
- multi-item experimental scales with items selected after multivariate testing of suicide risk factors;
- multivariate modelling other than machine learning (using statistically optimised risk models, for example classification plots generated by multiple regressions techniques); and
- machine learning (defined as any advanced experimental technique utilising computerised learning).

Where more than one type of suicide prediction model was described in a publication (as defined above) both suicide prediction models were included. When more than one suicide prediction model was reported using a single type of model (as defined above) only the model with the highest OR was used. When more than one population was reported in a single publication (such as men and women) both suicide prediction models were included.

Four continuous moderator variables were collected to investigate between-study heterogeneity in ORs:

- the number of included suicide risk factors in the suicide prediction models (because risk models that rely on more detailed information might be more accurate);
- the number of potential suicide risk factors (because studies considering more factors initially might then include more factors in the suicide prediction model);
- the mean length of follow-up (because studies with longer follow-up are less likely to misclassify eventual suicides); and
- publication year (because prediction might have improved over time with the introduction of more advanced types of suicide prediction model such as machine learning).

The number of potential suicide risk factors examined was often reported in the primary research paper or in an associated publication. Otherwise the number of potential risk factors was ascertained by counting all the independent variables listed in the paper. The number and nature of suicide risk factors included in the suicide prediction models was clearly documented in almost every study.

The study diagnostic group (schizophrenia spectrum, affective disorder, mixed diagnosis or other) and the research setting (general population/non-psychiatric care, after any form of self-harm or suicide attempt, specialist mental healthcare, discharged psychiatric in-patients, current psychiatric in-patients and in correctional settings) were collected as potential moderators of the strength of suicide prediction.

### Assessment of strength of reporting in primary studies

The strength of reporting in the primary studies was assessed using a 0–6 point scale derived from the Newcastle-Ottawa scale for the assessment of reporting strength of non-randomised studies^15^ and adapted from a scale used in two earlier meta-analyses of suicide prediction models.^7,14^ One point was assigned for each of the following items:

- cohort study (rather than a controlled study);
- gender-matched cohorts or controls;
- individuals recruited from a defined geographic area;
- suicide risk factors recorded prospectively (rather than by examination of medical records);
- suicides ascertained using an external mortality database (rather than suicide as determined by the researchers); and
- used ‘persons’ rather than clinical contacts as the denominator.

Studies with a score of 4 or more were considered to have stronger reporting and to be at lower risk of bias.

### Data synthesis

A random-effects model was used to calculate the pooled effect size in the form of ORs using Comprehensive Meta-Analysis.^16^ Secondary outcomes included the sensitivity, specificity and positive predictive value. Between-study heterogeneity was examined using the *I*^2^ and *Q*-value statistics. A meta-analytic estimate of the receiver operator curve and area under the curve was calculated using Meta-DiSc.^17^ The possibility of publication bias was assessed using Egger's regression^18^ and was quantified using Duval and Tweedie's trim and fill method.^19^

The analysis was conducted at the level of suicide prediction model but a sensitivity analysis was conducted using publication as the unit of analysis. Between-group heterogeneity was examined using a mixed-effects model without assuming a common within-study variance and the significance of between-group heterogeneity was determined with *Q*-value statistics. Between-group analyses were performed to examine the moderation of OR according to: (a) the type of the suicide prediction model; (b) cohort versus control design; (c) diagnostic group; (d) research setting; and (e) strength of reporting items.

Random-effects meta-regression (method of moments) was used to examine whether continuous moderators were associated with between-study heterogeneity in ORs. Multiple meta-regression was used to assess the independence of the associations between the moderator of machine learning, the number of included suicide risk factors and the OR.

### Ethical approval

No ethical approval was required.

## Results

### Searches and data extraction

The searches identified 86 studies^20–105^ reporting 102 samples of people who were categorised as being at high suicide risk using an exploratory suicide prediction model (see Supplementary Material Table S1: table of included studies reporting exploratory suicide prediction models; Supplementary Material Table S2: data used in the meta-analysis and ratings of strength of reporting in primary research; available at https://doi.org/10.1192/bjo.2020.162). There were four disagreements about the selection of studies that were resolved by consensus. An initial data extraction from a subset of studies resulted in disagreements in almost one-third of the effect-size data because of the selection of different cut-off points and different suicide prediction models. A second independent data extraction of the full data-set resulted in disagreements about effect-size data in 10 of the 102 (10%) suicide prediction models, all of which were resolved by further examination. Re-examination resolved 198 differences in 1428 (14%) moderator or reporting strength data points.

The included papers examined 20 210 411 people of whom 106 902 died by suicide. The earliest paper was published in 1966 and the median publication year was 2007. The total number of potential suicide risk factors examined was 12 242 (mean per model = 135, s.d. = 387) after excluding the samples that used clinical judgement (in which the number of potential and included suicide risk factors could not be ascertained) and a single machine-learning study that used 8071 predictor variables.^62^ The total number of included suicide risk factors in the suicide prediction models could not be ascertained exactly because some machine-learning studies did not clarify this precisely, but there were at least 777 (mean per model 8.7, s.d. = 9.9) of which 598 could be identified and tabulated (Supplementary Material Table S1, available at https://doi.org/10.1192/bjo.2020.162). The median reported OR was 8.0, the first quartile was 3.8, the third quartile was 19.2 and the range was 1.05–297.

### Meta-analysis

**Fig. 2:**
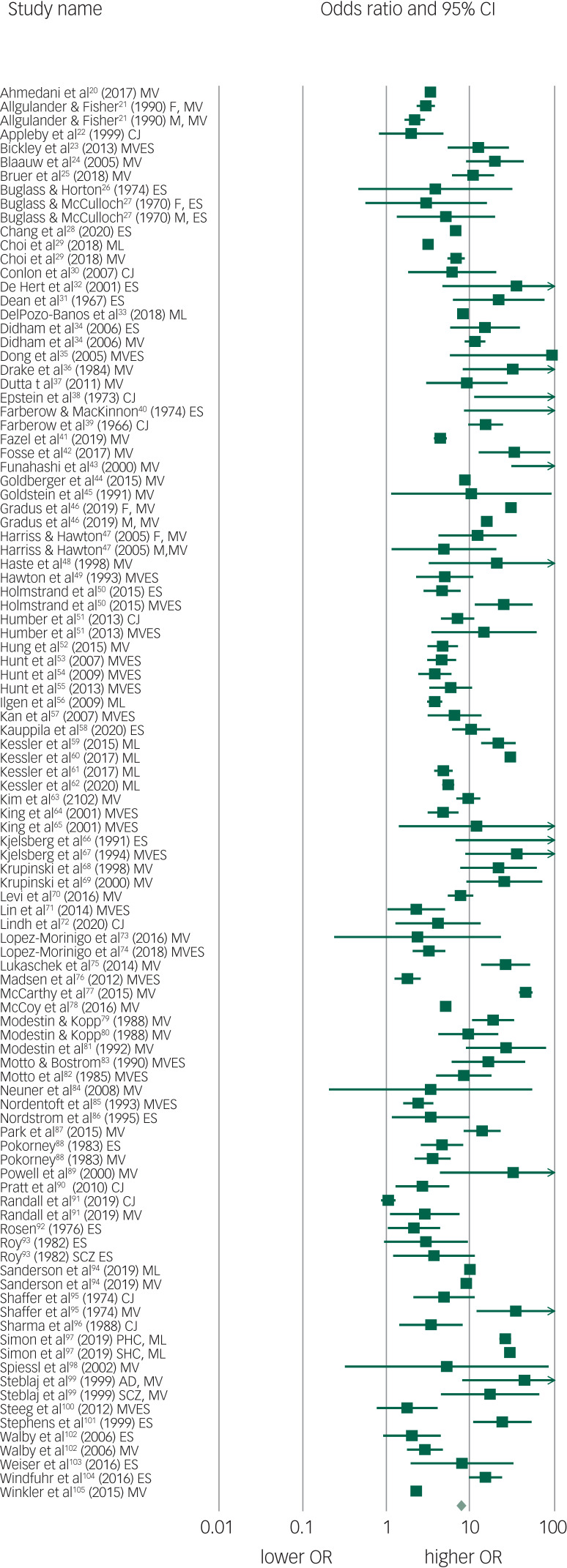
Forest plot of suicide prediction models. CJ, clinical judgement; MV, multivariate model; MVES, experimental scale based on multivariate analysis; ES, experimental scale based no bivariate analysis; ML, machine learning; M, male; F, female; AD, affective disorder; SCZ, schizophrenia spectrum; PHC, primary health care; SHC, secondary health care; OR, odds ratio.

**Fig. 3:**
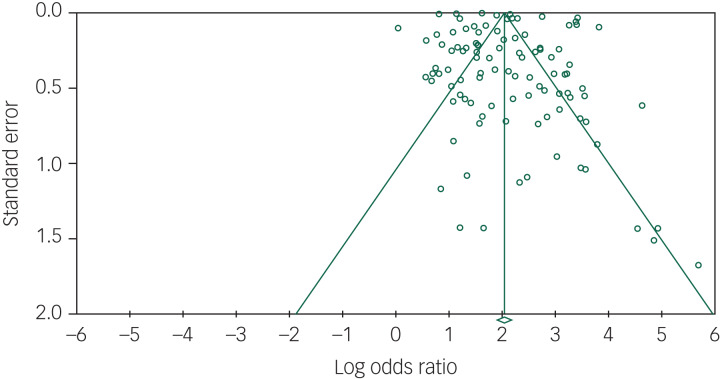
Funnel plot of standard error by log odds ratio of suicide prediction models.

**Fig. 4:**
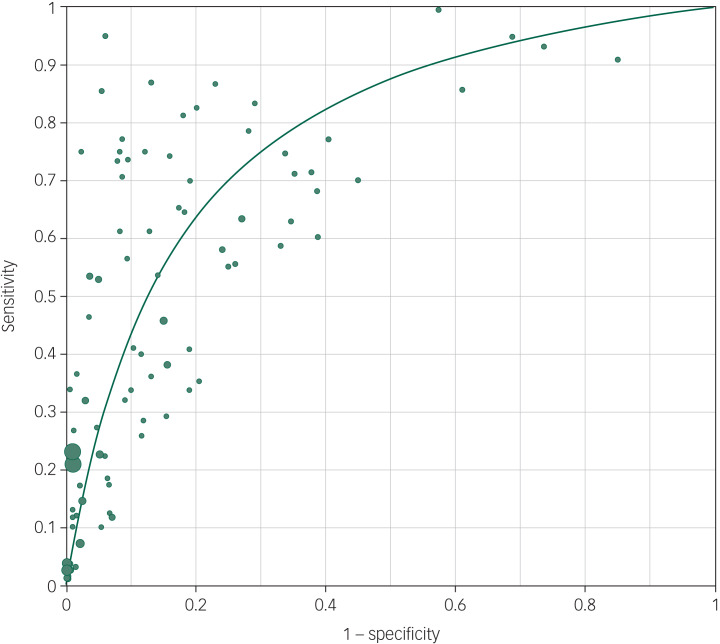
Receiver operating curve of exploratory suicide prediction models.

The pooled OR of 102 suicide prediction models was 7.7 (95% CI 6.7–8.8; 95% prediction interval, 2.5–23.9) (Fig. 2) indicating a strong effect size.^106^ There was very high between-study heterogeneity (*I*^2^ = 99.5, *Q*-value 20 435, *P* < 0.001). An analysis using the 86 publications as the unit of analysis had a 2.5% lower OR of 7.5 (95% CI 6.5–8.7). There was some evidence of publication bias in favour of studies reporting a higher OR using Egger's test (intercept 2.94, *t*-value 1.99, two-tailed *P* = 0.05) (Fig. 3). Duval and Tweedie's trim and fill method identified 12 hypothetically missing studies with lower ORs and estimated an 11.5% lower OR of 6.8 (95% CI 6.0–7.8). The models achieved their ORs using differing trade-offs between sensitivity and specificity (Fig. 4). The pooled sensitivity of a high-risk prediction was 44% (*n* = 88 studies, 95% CI 37–50%, *I*^2^ = 99.5) and the pooled specificity of a lower-risk prediction was 84% (*n* = 88 studies, 95% CI 79–88%, *I*^2^ = 99.9). The pooled area under the curve was 0.79. The positive predictive value was 2.8% (95% CI 1.8–4.3%, *I*^2^ = 98.8) among 36 cohort studies.

**Fig. 5:**
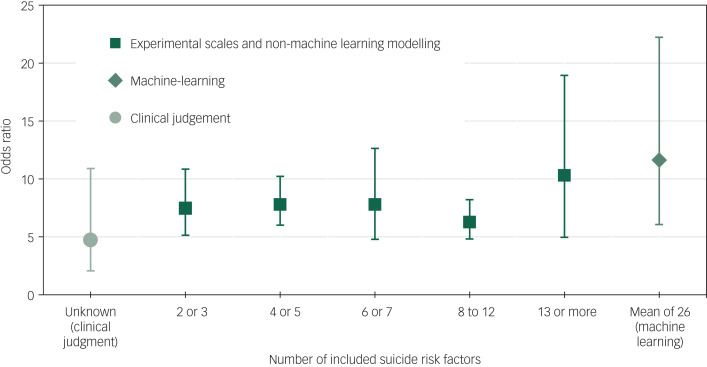
Clinical judgement, machine learning and the number of included risk variables in suicide prediction models.

Suicide prediction models that used experimental scales derived from bivariate or multivariate testing performed better than clinical judgement. Multivariate modelling and machine learning achieved a higher OR than experimental scales. A seemingly large difference in ORs according to these types of suicide prediction model did not reach clinical significance (Table 1 and Fig. 5). The number of included suicide risk factors in the suicide prediction model (excluding ten clinical judgement samples and three models in which the number of included suicide risk variables was not reported) was significantly associated with the ORs, but this moderator was not significantly associated with the ORs among the 79 non-machine-learning, non-clinical judgement studies (Table 2). Machine-learning studies included a mean of 26 risk suicide factors whereas non-machine-learning studies (excluding clinical judgement studies that were not included in any analyses of the number of risk factors) incorporated a mean of 6.5 suicide risk factors. A multiple meta-regression found that neither machine learning (coefficient 0.21, 95% CI −0.50 to 0.91, *Z*-value = 0.57, *P* = 0.56) or the number of included suicide risk factors (coefficient 0.01, 95% CI −0.01 to 0.04, *Z*-value = 1.42, *P* = 0.15) made an independent contribution to heterogeneity in ORs and together explained about one tenth of the between-study heterogeneity (*Q* = 5.56, d.f. = 2, *P* = 0.06, R-square analogue 10.1%).

**Table 1:** Meta-analysis of study methods suicide and the strength of prediction models

	Samples, *n*	Odds ratio	Lower limit 95% CI	Upper limit 95% CI	*I*^2^	Heterogeneity		
						*Q*-value	d.f. (*Q*)	*P*
Pooled estimate								
Random effects	102	7.7	6.7	8.8	99.5			
Machine learning compared with other methods of suicide prediction models								
Other methods	90	7.0	6.1	8.1	99.1	2.2	1	0.14
Machine learning	12	11.6	6.0	22.3	99.9			
Study design								
Case–control	60	10.1	8.2	12.4	98.2	18.9	1	<0.0001
Cohort	42	5.5	4.5	6.6	99.7			
Matching for gender								
No matching	55	7.0	6.0	8.3	99.6	2.5	1	0.11
Matching	47	8.9	7.0	11.5	97.9			
Defined catchment area								
Not defined	43	10.2	7.7	13.4	97.7	5.4	1	0.02
Defined catchment	59	6.7	5.4	8.4	99.7			
Pre-collected risk factors								
Chart review	39	9.2	6.8	12.4	82.1	1.7	1	0.19
Pre-collected	63	7.3	6.2	8.6	99.7			
Denominator is ‘persons’								
Clinical contact	8	14.1	7.7	25.8	98.7	4.6	1	0.03
Persons	94	7.1	6.2	8.1	99.5			
Suicides ascertained by external mortality database								
Local data	30	14.0	8.7	22.6	96.3	8.6	1	0.003
External data	72	6.6	5.7	7.7	99.6			
Overall strength of reporting								
Less strong reporting	48	12.8	9.0	18.2	97.6	17.4	1	<0.0001
Stronger reporting	54	5.6	4.7	6.6	99.7			
Type of suicide prediction model								
Clinical judgement	10	4.7	2.1	10.9	94.1	8.5	4	0.07
Experimental scale based on bivariate statistics	20	6.9	5.0	9.5	72.6			
Experimental scale based on multivariate statistics	19	5.6	4.0	8.0	80.5			
Multivariate model (other than machine learning)	41	9.0	7.3	11.1	99.6			
Machine-learning model	12	11.6	6.0	22.3	99.9

**Table 2:** Meta-regression of continuous moderator variables and the strength of prediction models

	Samples, *n*	Coefficient	s.e.	Lower limit 95% CI	Upper limit 95% CI	*Z*-value	*P*
Number included suicide risk factors	89	0.02	0.01	0.003	0.04	2.3	0.02
Number of included suicide risk factors excluding machine-learning studies	79	0.01	0.02	−0.03	0.05	0.52	0.60
Number of potential suicide risk factors	92	0.0001	0.0001	−0.0001	0.0002	0.45	0.65
Mean length of follow-up	84	0.0003	0.0009	−0.0015	0.002	0.36	0.72
Publication year	102	−0.002	0.007	−0.015	0.011	−0.33	0.74

Cohort studies, those conducted in geographically defined catchment areas and studies that used external suicide mortality data had a lower pooled OR, respectively, than controlled studies, those that were based on a hospital or health service sample and those that assessed suicide mortality using local data (Table 1). Groups of studies that were matched for gender and those that collected research data prospectively had similar pooled ORs to those that were, respectively, not gender matched or that extracted the research data from medical records (Table 1).

The group of studies that examined suicides based on clinic contacts rather than individuals reported a higher pooled OR (Table 1). Suicide prediction models performed better among cohorts of people with schizophrenia, in primary health/general population samples and among psychiatric in-patients. Suicide prediction models performed less well among cohorts of patients who had presented with suicide attempts or self-harm (Table 3).

**Table 3:** Meta-analysis of diagnostic groups and research settings and the strength of prediction models

	Samples, *n*	Odds ratio	Lower limit 95% CI	Upper limit 95% CI	*I*^2^	Between-group heterogeneity		
						*Q*-value	d.f. (*Q*)	*P*
Diagnostic group								
Affective disorders	8	10.4	7.1	15.1	31.2	11.4	2	0.003
Schizophrenia spectrum	12	17.2	9.5	31.2	66.6			
Mixed diagnosis and other diagnoses^a^	82	7.0	6.0	8.1	99.6			
Study setting								
Primary health/general population	24	10.9	8.7	13.8	99.8	71.4	5	<0.0001
Specialist mental healthcare	15	5.5	2.7	11.2	99.6			
Post self-harm	12	2.9	2.3	3.7	32.3			
Post psychiatric discharge	25	8.6	5.7	13.0	99.7			
Current psychiatric in-patients	22	10.5	6.8	16.2	84.9			
Correctional settings	4	8.0	3.5	18.5	78.2			

a. Includes one sample of patients with borderline personality disorder and one sample of people with epilepsy.

Meta-regression found no evidence that the year of publication or length of follow-up contributed to between-study heterogeneity (Table 2). The number of potential suicide risk factors did not contribute to between-sample heterogeneity (Table 2). Further, machine learning made no independent contribution to between-sample heterogeneity in various *post hoc* multiple meta-regression that included the methodological variable of whether the study used people or clinical contacts in the denominator and other indicators of reporting strength. (For the full data-set used in the study including effect-size data, moderators and strength of reporting data see Supplementary Material Table S2.)

### Suicide risk factors in the suicide prediction models

A previous suicide attempt or any form of self-harm was the most common included suicide risk factor that was present in 68 of the 102 suicide prediction models. This was followed by depressive diagnosis or symptoms (*n* = 50), alcohol or substance use (*n* = 34), suicidal ideation (*n* = 31), previous psychiatric hospital admission (*n* = 24), male gender (*n* = 20), psychotropic medication (*n* = 20), physical comorbidity (*n* = 19), psychiatric diagnosis (*n* = 19), psychotic symptoms (*n* = 19), older age (*n* = 17), previous psychiatric care (*n* = 17), adverse life events (*n* = 12), family history of mental disorder or suicide (*n* = 12), childhood or developmental adversity (*n* = 11), ethnicity (*n* = 9), anxiety disorder (*n* = 8), forensic history (*n* = 7), housing problems (*n* = 7), bipolar disorder (*n* = 7), personality disorder (*n* = 7), schizophrenia spectrum disorder (*n* = 7), problems with psychiatric discharge (*n* = 6), longer psychiatric hospitalisation (*n* = 6), problems with rapport (*n* = 6), current financial stress (*n* = 5) and involuntary psychiatric treatment (*n* = 5). Some risk factors were included with opposing effect sizes, for example both employment and unemployment were included in (*n* = 4) suicide prediction models, and being divorced or not married (*n* = 6) and being married (*n* = 3) appeared in suicide prediction models.

## Discussion

This synthesis of a large and representative sample of exploratory suicide prediction models found a strong statistical association between being allocated to the highest suicide risk category and suicide. The meta-analyses of sensitivity and specificity suggests that under half of all suicides might be anticipated by suicide prediction models, with non-suicide being incorrectly predicted in more than one in ten cases. Meta-analyses of a subsample of cohort studies suggested that 34 in 35 suicide predictions are likely to be false positive predictions.

The first aim of our study was to assess whether machine-learning suicide prediction models had stronger prediction than studies using other types of suicide prediction. The second aim was to determine whether suicide prediction models with a larger number of included suicide risk factors had stronger prediction than suicide prediction models with fewer factors. While we had conceptualised these as separate questions, machine-learning studies included an average of four times the number of suicide risk factors than other types of suicide prediction model. When all the studies (except clinical judgement studies) were included, the number of included suicide risk factors was positively associated with the OR in line with our hypothesis. However, when machine-learning studies were excluded or when machine learning was used as a covariable in a multiple meta-regression, there was no association between the number of included suicide risk variables and the OR. This is in line with our findings in longitudinal suicide studies^7^ and among samples of psychiatric in-patients^14^ that models with more risk factors do not necessarily produce greater predictive strength.

This result is of interest because it challenges the traditional notion that considering more risk factors will necessarily improve suicide risk assessment. Further, the result is consistent with a key difference between machine learning and other types of suicide prediction model in that machine learning can potentially recognise the context of factors, such that factors with no overall association with suicide might be included if they confer risk in some contexts but are protective in others. Further research might show that machine learning can improve suicide risk prediction because it can recognise the context and non-linear relationships between risk factors.

However, in this data-set even though the effect size derived from machine learning was moderately stronger than other suicide prediction models, the *P*-value was below statistical significance. A future meta-analysis with more machine-learning studies may have a different result. Although this is encouraging for future machine-learning research, we urge some caution. In particular, the machine-learning studies we included had other study characteristics that might have inflated their results. In addition to examining a large number of potential suicide risk factors increasing the possibility of chance associations,^46,59,97^ some machine-learning studies examined clinical contacts rather than people, resulting in possible oversampling of suicides if they were more frequent attenders.^60,77,97^ Other machine-learning studies oversimplified the spectrum of non-suicide presentations by selective sampling and overweighting of a small proportion of controls.^60,61^ Still other machine-learning studies developed a large number of synthetic or compound potential suicide risk factors (for example alcohol use by various age and gender groups) increasing the risk of chance associations.^46,97^ We conclude that the case for machine learning as a statistically superior method of suicide prediction model is not yet conclusive.

Our findings also present an interesting conundrum for the clinician: although increasing the number of included risk factors will improve the strength of machine-learning programs, clinicians who use fewer risk factors to identify suicide risk are likely to do as well as those who use more complex risk models.

### Limitations to the generalisability of the pooled estimates

The very high between-study heterogeneity in effect size means that our pooled estimates may not be generalisable. Moreover, it is likely that the pooled effect size we report would not be as strong in replication/validation studies, given our results are based on exploratory studies in which the suicide prediction models were retro-fitted to suicide and survivor data.^107^ The potential for chance findings in the primary research and therefore our pooled ORs is illustrated by the threshold *P*-value of 0.05 for the inclusion of a suicide risk factor and the large number of potential suicide risk factors examined. Of the over 12 000 potential suicide risk factors examined in the primary research, 1 in 20, or over 600 (77% of all the 777 included risk factors) might have been included by chance with a threshold of *P* = 0.05. This may not have had a great effect on the ultimate strength of the pooled effect size because the number of potential risk factors was not associated with the ultimate effect size and the number of included suicide risk factors (possibly including some chance associations) did not increase the strength of the predictions in non-machine-learning models. Notably, studies that used two or three included suicide risk factors performed just as well as studies that used more variables. Other cautions about the strength of the effect size reported flow from the statistical evidence of publication bias in favour of studies with a larger OR as well as the lower OR in cohort studies and those with generally stronger methods.

### Other findings

We found evidence that suicide prediction models work better in the general community/non-mental health settings and in current in-patient settings. Although these finding were incidental and might not be replicated, it is possible that suicide risk factors are more meaningful in settings where the risk factors are less prevalent (such as a psychiatric diagnosis in the general community) and when more accurate and detailed risk profiles are available (such as in a hospital). Suicide prediction models seemed to perform less well after suicide attempts or self-harm. The most obvious reason for this is that suicide attempts or self-harm, which were the single most commonly included suicide risk factor, cannot be used in the suicide prediction model if both the suicides and survivors have this risk factor.

### Implications

Machine learning makes it possible for vast amounts of information to be modelled to predict suicide risk, but to do this, much more information, including those for economic, social and network factors not traditionally examined in a clinical setting, might be needed. It is possible that machine learning will ultimately produce better suicide prediction models that are more clinically applicable, as well as better reflecting our intuitive knowledge about the complex social, cultural and personal factors that contribute to an individual's suicide. Alternatively, it might be that suicide is fundamentally unpredictable, with most of the uncertainty about suicide being aleatory rather than epistemic and therefore not very amenable to prediction.^8^ In the meantime, we suggest that future machine-learning studies should focus on clinically relevant input variables, use people rather than clinical contacts as the denominator, and should examine ways of combining computational results with broader clinical considerations.

Although the potential of machine learning in suicide prediction remains uncertain, it should not be forgotten that the utility of any suicide prediction model depends on its clinical application and not its effect size or *P*-value. Even a suicide prediction model producing a strong statistical association may not be useful if there are no rational interventions that can be provided for people who are predicted to die by suicide, remembering the vast majority of predictions are false positives, or if there are no rational interventions that should be withheld from patients classified as lower risk, among whom many suicides might occur. Ultimately the utility of a suicide prediction model should not be evaluated by its statistical strength or perceived suitability to guide interventions, but should be judged by its contribution to a reduction in suicide mortality. Until such time as the use of any suicide prediction model has been shown to reliably reduce suicide, our clinical advice is to focus on understanding and caring for the person in front of us, including by treating any modifiable risk factors, irrespective of estimations of any overall suicide risk category.

## Data Availability

All the data upon which the study is based has been submitted in the supplementary material.

## Supplementary material

For supplementary material accompanying this paper visit http://dx.doi.org/10.1192/bjo.2020.162. click here to view supplementary material

## References

[1] Franklin JC, Ribeiro JD, Fox KR, Bentley KH, Kleiman EM, Huang X, Risk factors for suicidal thoughts and behaviors: a meta-analysis of 50 years of research. Psychol Bull 2017; 143: 187–232. 27841450 10.1037/bul0000084

[2] Ribeiro JD, Franklin JC, Fox KR, Bentley KH, Kleiman EM, Chang BP, Self-injurious thoughts and behaviors as risk factors for future suicide ideation, attempts, and death: a meta-analysis of longitudinal studies. Psychol Med 2016; 46: 225–36. 26370729 10.1017/S0033291715001804 PMC4774896

[3] Chan MK, Bhatti H, Meader N, Stockton S, Evans J, O'Connor RC, Predicting suicide following self-harm: systematic review of risk factors and risk scales. Br J Psychiatry 2016; 209: 277–83. 27340111 10.1192/bjp.bp.115.170050

[4] Carter G, Milner A, McGill K, Pirkis J, Kapur N, Spittal MJ. Predicting suicidal behaviours using clinical instruments: systematic review and meta-analysis of positive predictive values for risk scales. Br J Psychiatry 2017; 210: 387–95. 28302700 10.1192/bjp.bp.116.182717

[5] Runeson B, Odeberg J, Pettersson A, Edbom T, Jildevik Adamsson I, Waern M. Instruments for the assessment of suicide risk: a systematic review evaluating the certainty of the evidence. PLoS One 2017; 12: e0180292. 28723978 10.1371/journal.pone.0180292 PMC5517300

[6] Belsher BE, Smolenski DJ, Pruitt LD, Bush NE, Beech EH, Workman DE, Prediction models for suicide attempts and deaths: a systematic review and simulation. JAMA Psychiatry 2019; 76: 642–51. 30865249 10.1001/jamapsychiatry.2019.0174

[7] Large M, Kaneson M, Myles N, Myles H, Gunaratne P, Ryan C. Meta-analysis of longitudinal cohort studies of suicide risk assessment among psychiatric patients: heterogeneity in results and lack of improvement over time. PLoS One 2016; 11: e0156322. 27285387 10.1371/journal.pone.0156322 PMC4902221

[8] Large M, Galletly C, Myles N, Ryan CJ, Myles H. Known unknowns and unknown unknowns in suicide risk assessment: evidence from meta-analyses of aleatory and epistemic uncertainty. BJPsych Bull 2017; 41: 160–3. 28584653 10.1192/pb.bp.116.054940 PMC5451650

[9] Wilkinson J, Arnold K, Murray E, van Smeden M, Carr K, Sippy R, Time to reality check the promises of machine learning-powered precision medicine. Lancet Digital Health 2020; 2: E677–80. 10.1016/S2589-7500(20)30200-4 PMC9060421 33328030

[10] McHugh CM, Large MM. Can machine-learning methods really help predict suicide? Curr Opinion Psychiatry 2020; 33: 369–74. 10.1097/YCO.0000000000000609 32250986

[11] Liberati A, Altman DG, Tetzlaff J, Mulrow C, Gøtzsche PC, Ioannidis JP, The PRISMA statement for reporting systematic reviews and meta-analyses of studies that evaluate health care interventions: explanation and elaboration. J Clin Epidemiol 2009; 62: e1–34. 19631507 10.1016/j.jclinepi.2009.06.006

[12] Stroup DF, Berlin JA, Morton SC, Olkin I, Williamson GD, Rennie D, Meta-analysis of observational studies in epidemiology: a proposal for reporting. Meta-analysis of Observational Studies in Epidemiology (MOOSE) group. JAMA 2000; 283: 2008–12. 10789670 10.1001/jama.283.15.2008

[13] McHugh CM, Corderoy A, Ryan CJ, Hickie IB, Large MM. Association between suicidal ideation and suicide: meta-analyses of odds ratios, sensitivity, specificity and positive predictive value. BJPsych Open 2019; 5: e1. 30702058 10.1192/bjo.2018.88 PMC6401538

[14] Large M, Myles N, Myles H, Corderoy A, Weiser M, Davidson M, Suicide risk assessment among psychiatric inpatients: a systematic review and meta-analysis of high-risk categories. Psychol Med 2018; 48: 1119–27. 28874218 10.1017/S0033291717002537

[15] Wells GA, Shea B, O'Connnell D, Peterson J, Welch V, Losos M, *The Newcastle-Ottawa Scale (NOS) for Assessing the Quality of Nonrandomised Studies in Meta-Analyses*. The Ottowa Hospital Research Institute, 2013 (http://www.ohri.ca/programs/clinical_epidemiology/oxford.asp).

[16] Borenstein M, Hedges L, Higgins J, Rothstein H. Comprehensive Meta-Analysis Version 3. Biostat, 2013.

[17] Zamora J, Abraira V, Muriel A, Khan K, Coomarasamy A. Meta-DiSc: a software for meta-analysis of test accuracy data. BMC Med Res Methodol 2006; 6: 31. 16836745 10.1186/1471-2288-6-31 PMC1552081

[18] Egger M, Davey Smith G, Schneider M, Minder C. Bias in meta-analysis detected by a simple, graphical test. BMJ 1997; 315: 629–34. 9310563 10.1136/bmj.315.7109.629 PMC2127453

[19] Duval S, Tweedie R. Trim and fill: a simple funnel-plot-based method of testing and adjusting for publication bias in meta-analysis. Biometrics 2000; 56: 455–63. 10877304 10.1111/j.0006-341x.2000.00455.x

[20] Ahmedani BK, Peterson EL, Hu Y, Lynch F, Lu CY, Waitzfelder BE, Major physical health conditions and risk of suicide. Am J Prev Med 2017; 53: 308–15. 28619532 10.1016/j.amepre.2017.04.001 PMC5598765

[21] Allgulander C, Fisher LD. Clinical predictors of completed suicide and repeated self-poisoning in 8895 self-poisoning patients. Eur Arch Psychiatry Neurol Sci 1990; 239: 270–6. 2138552 10.1007/BF01738583

[22] Appleby L, Dennehy JA, Thomas CS, Faragher EB, Lewis G. Aftercare and clinical characteristics of people with mental illness who commit suicide: a case-control study. Lancet 1999; 353: 1397–400. 10227220 10.1016/S0140-6736(98)10014-4

[23] Bickley H, Hunt IM, Windfuhr K, Shaw J, Appleby L, Kapur N. Suicide within two weeks of discharge from psychiatric inpatient care: a case-control study. Psychiatr Serv 2013; 64: 653–9. 23545716 10.1176/appi.ps.201200026

[24] Blaauw E, Kerkhof AJ, Hayes LM. Demographic, criminal, and psychiatric factors related to inmate suicide. Suicide Life Threat Behav 2005; 35: 63–75. 15843324 10.1521/suli.35.1.63.59268

[25] Bruer RA, Rodway-Norman M, Large M. Closer to the truth: admission to multiple psychiatric facilities and an inaccurate history of hospitalization are strongly associated with inpatient suicide. Can J Psychiatry 2018; 63: 748–56. 29685070 10.1177/0706743718772519 PMC6299186

[26] Buglass D, Horton J. A scale for predicting subsequent suicidal behaviour. Br J Psychiatry 1974; 124: 573–8. 4850288 10.1192/bjp.124.6.573

[27] Buglass D, McCulloch JW. Further suicidal behaviour: the development and validation of predictive scales. Br J Psychiatry 1970; 116: 483–91. 5460558 10.1192/bjp.116.534.483

[28] Chang CF, Yeh MK, Chien WC, Chung CH, Li TT, Lai EC. Interactions between psychiatric and physical disorders and their effects on the risks of suicide: a nested case-control study. Ann N Y Acad Scis 2020; 1462: 79–91. 10.1111/nyas.14216 31495960

[29] Choi SB, Lee W, Yoon JH, Won JU, Kim DW. Ten-year prediction of suicide death using Cox regression and machine learning in a nationwide retrospective cohort study in South Korea. J Affect Disord 2018; 231: 8–14. 29408160 10.1016/j.jad.2018.01.019

[30] Conlon L, Garland M, Prescott P, Mannion L, Leonard M, Fahy TJ. Psychiatric aftercare and suicide risk: a case-control study using blind rating. Arch Suicide Res 2007; 11: 291–5. 17558614 10.1080/13811110701404021

[31] Dean RA, De Cook R, Maley RF, Miskimins W, Wilson LT. Prediction of suicide in a psychiatric hospital. J Clin Psychol 1967; 23: 296–301. 6082115 10.1002/1097-4679(196707)23:3<296::aid-jclp2270230304>3.0.co;2-7

[32] De Hert M, McKenzie K, Peuskens J. Risk factors for suicide in young people suffering from schizophrenia: a long-term follow-up study. Scizophren Res 2001; 47: 127–34. 10.1016/s0920-9964(00)00003-7 11278129

[33] DelPozo-Banos M, John A, Petkov N, Mark Berridge D, Southern K, LLoyd K, Using neural networks with routine health records to identify suicide risk: feasibility study. JMIR Ment Health 2018; 5: e10144. 29934287 10.2196/10144 PMC6035342

[34] Didham R, Dovey S, Reith D. Characteristics of general practitioner consultations prior to suicide: a nested case-control study in New Zealand. N Z Med J 2006; 119: U2358. 17195851

[35] Dong JY, Ho TP, Kan CK. A case-control study of 92 cases of in-patient suicides. J Affect Disord 2005; 87: 91–9. 15967234 10.1016/j.jad.2005.03.015

[36] Drake RE, Gates C, Cotton PG, Whitaker A. Suicide among schizophrenics. Who is at risk? J Nerv Ment Dis 1984; 172: 613–7. 6481346 10.1097/00005053-198410000-00004

[37] Dutta R, Murray RM, Allardyce J, Jones PB, Boydell J. Early risk factors for suicide in an epidemiological first episode psychosis cohort. Scizophren Res 2011; 126: 11–9. 10.1016/j.schres.2010.11.021 21183318

[38] Epstein LC, Thomas CB, Shaffer JW, Perlin S. Clinical prediction of physician suicide based on medical student data. J Nerv Ment Dis 1973; 156: 19–29. 4690309 10.1097/00005053-197301000-00002

[39] Farberow NL, Shneidman ES, Neuringer C. Case history and hospitalization factors in suicides of neuropsychiatric hospital patients. J Nerv Ment Dis 1966; 142: 32–44. 5931169 10.1097/00005053-196601000-00006

[40] Farberow NL, MacKinnon D. A suicide prediction schedule for neuropsychiatric hospital patients. J Nerv Ment Dis 1974; 158: 408–19. 4841286 10.1097/00005053-197406000-00003

[41] Fazel S, Wolf A, Larsson H, Mallett S, Fanshawe TR. The prediction of suicide in severe mental illness: development and validation of a clinical prediction rule (OxMIS). Transl Psychiatry 2019; 9: 98. 30804323 10.1038/s41398-019-0428-3 PMC6389890

[42] Fosse R, Ryberg W, Carlsson MK, Hammer J. Predictors of suicide in the patient population admitted to a locked-door psychiatric acute ward. PLoS One 2017; 12: e0173958. 28301590 10.1371/journal.pone.0173958 PMC5354397

[43] Funahashi T, Ibuki Y, Domon Y, Nishimura T, Akehashi D, Sugiura H. A clinical study on suicide among schizophrenics. Psychiatry Clin Neurosci 2000; 54: 173–9. 10803812 10.1046/j.1440-1819.2000.00655.x

[44] Goldberger N, Haklai Z, Pugachova I, Levav I. Suicides among persons with psychiatric hospitalizations. Isr J Psychiatry Relat Sci 2015; 52: 25–31. 25841107

[45] Goldstein RB, Black DW, Nasrallah A, Winokur G. The prediction of suicide. Sensitivity, specificity, and predictive value of a multivariate model applied to suicide among 1906 patients with affective disorders. Arch Gen Psychiatry 1991; 48: 418–22. 2021294 10.1001/archpsyc.1991.01810290030004

[46] Gradus JL, Rosellini AJ, Horvath-Puho E, Street AE, Galatzer-Levy I, Jiang T, Prediction of sex-specific suicide risk using machine learning and single-payer health care registry data from Denmark. JAMA Psychiatry 2019; 77: 25–34. 10.1001/jamapsychiatry.2019.2905 PMC6813578 31642880

[47] Harriss L, Hawton K. Suicidal intent in deliberate self-harm and the risk of suicide: the predictive power of the Suicide Intent Scale. J Affect Disord 2005; 86: 225–33. 15935242 10.1016/j.jad.2005.02.009

[48] Haste F, Charlton J, Jenkins R. Potential for suicide prevention in primary care? An analysis of factors associated with suicide. Br J Gen Pract 1998; 48: 1759–63. 10198484 PMC1313268

[49] Hawton K, Fagg J, Platt S, Hawkins M. Factors associated with suicide after parasuicide in young people. BMJ 1993; 306: 1641–4. 8324431 10.1136/bmj.306.6893.1641 PMC1678090

[50] Holmstrand C, Bogren M, Mattisson C, Bradvik L. Long-term suicide risk in no, one or more mental disorders: the Lundby Study 1947–1997. Acta Psychiatr Scand 2015; 132: 459–69. 26402416 10.1111/acps.12506 PMC5054879

[51] Humber N, Webb R, Piper M, Appleby L, Shaw J. A national case-control study of risk factors for suicide among prisoners in England and Wales [corrected]. Soc Psychiatry Psychiatr Epidemiol 2013; 48: 1177–85. 23232691 10.1007/s00127-012-0632-4

[52] Hung GC, Kwok CL, Yip PS, Gunnell D, Chen YY. Predicting suicide in older adults – a community-based cohort study in Taipei City, Taiwan. J Affect Disord 2015; 172: 165–70. 25451412 10.1016/j.jad.2014.09.037

[53] Hunt IM, Kapur N, Webb R, Robinson J, Burns J, Turnbull P, Suicide in current psychiatric in-patients: a case-control study The National Confidential Inquiry into Suicide and Homicide. Psychol Med 2007; 37: 831–7. 17306045 10.1017/S0033291707000104

[54] Hunt IM, Kapur N, Webb R, Robinson J, Burns J, Shaw J, Suicide in recently discharged psychiatric patients: a case-control study. Psychol Med 2009; 39: 443–9. 18507877 10.1017/S0033291708003644

[55] Hunt IM, Bickley H, Windfuhr K, Shaw J, Appleby L, Kapur N. Suicide in recently admitted psychiatric in-patients: a case-control study. J Affect Disord 2013; 144: 123–8. 22871533 10.1016/j.jad.2012.06.019

[56] Ilgen MA, Downing K, Zivin K, Hoggatt KJ, Kim HM, Ganoczy D, Exploratory data mining analysis identifying subgroups of patients with depression who are at high risk for suicide. J Clin Psychiatry 2009; 70: 1495–500. 20031094 10.4088/JCP.08m04795 PMC3057750

[57] Kan CK, Ho TP, Dong JY, Dunn EL. Risk factors for suicide in the immediate post-discharge period. Soc Psychiatry Psychiatr Epidemiol 2007; 42: 208–14. 17268761 10.1007/s00127-006-0153-0

[58] Kauppila JH, Santoni G, Tao W, Lynge E, Jokinen J, Tryggvadóttir L, Risk factors for suicide after bariatric surgery in a population-based nationwide study in five nordic countries. Ann Surg [Epub ahead of print] 9 Jun 2020. Available from: 10.1097/SLA.0000000000004232. 32657942

[59] Kessler RC, Warner CH, Ivany C, Petukhova MV, Rose S, Bromet EJ, Predicting suicides after psychiatric hospitalization in US Army Soldiers: the army study to assess risk and resilience in servicemembers (Army STARRS). JAMA Psychiatry 2015; 72: 49–57. 25390793 10.1001/jamapsychiatry.2014.1754 PMC4286426

[60] Kessler RC, Hwang I, Hoffmire CA, McCarthy JF, Petukhova MV, Rosellini AJ, Developing a practical suicide risk prediction model for targeting high-risk patients in the Veterans health Administration. Int J Methods Psychiatr Res 2017; 26: e1575. 10.1002/mpr.1575 PMC5614864 28675617

[61] Kessler RC, Stein MB, Petukhova MV, Bliese P, Bossarte RM, Bromet EJ, Predicting suicides after outpatient mental health visits in the Army Study to Assess Risk and Resilience in Servicemembers (Army STARRS). Mol Psychiatry 2017; 22: 544–51. 27431294 10.1038/mp.2016.110 PMC5247428

[62] Kessler RC, Bauer MS, Bishop TM, Demler OV, Dobscha SK, Gildea SM, Using administrative data to predict suicide after psychiatric hospitalization in the veterans health administration system. Front Psychiatry 2020; 11: 390. 32435212 10.3389/fpsyt.2020.00390 PMC7219514

[63] Kim HM, Smith EG, Ganoczy D, Walters H, Stano CM, Ilgen MA, Predictors of suicide in patient charts among patients with depression in the Veterans Health Administration health system: importance of prescription drug and alcohol abuse. J Clin Psychiatry 2012; 73: e1269–75. 23140657 10.4088/JCP.12m07658

[64] King EA, Baldwin DS, Sinclair JM, Baker NG, Campbell MJ, Thompson C. The Wessex Recent In-Patient Suicide Study, 1. Case-control study of 234 recently discharged psychiatric patient suicides. Br J Psychiatry 2001; 178: 531–6. 11388969 10.1192/bjp.178.6.531

[65] King EA, Baldwin DS, Sinclair JM, Campbell MJ. The Wessex Recent In-Patient Suicide Study, 2. Case-control study of 59 in-patient suicides. Br J Psychiatry 2001; 178: 537–42. 11388970 10.1192/bjp.178.6.537

[66] Kjelsberg E, Eikeseth PH, Dahl AA. Suicide in borderline patients--predictive factors. Acta Psychiatr Scand 1991; 84: 283–7. 1950630 10.1111/j.1600-0447.1991.tb03145.x

[67] Kjelsberg E, Neegaard E, Dahl AA. Suicide in adolescent psychiatric inpatients: incidence and predictive factors. Acta Psychiatr Scand 1994; 89: 235–41. 8023689 10.1111/j.1600-0447.1994.tb01507.x

[68] Krupinski M, Fischer A, Grohmann R, Engel R, Hollweg M, Moller HJ. Risk factors for suicides of inpatients with depressive psychoses. Eur Arch Psychiatry Clin Neurosci 1998; 248: 141–7. 9728733 10.1007/s004060050031

[69] Krupinski M, Fischer A, Grohmann R, Engel RR, Hollweg M, Moller HJ. Schizophrenic psychoses and suicide in the clinic. Risk factors, psychopharmacologic treatment. Der Nervenarzt 2000; 71: 906–11. 11103366 10.1007/s001150050682

[70] Levi L, Werbeloff N, Pugachova I, Yoffe R, Large M, Davidson M, Has deinstitutionalization affected inpatient suicide? Psychiatric inpatient suicide rates between 1990 and 2013 in Israel. Scizophren Res 2016; 173: 75–8. 10.1016/j.schres.2016.03.007 26965744

[71] Lin SK, Hung TM, Liao YT, Lee WC, Tsai SY, Chen CC, Protective and risk factors for inpatient suicides: a nested case-control study. Psychiatry Res 2014; 217: 54–9. 24679994 10.1016/j.psychres.2014.03.008

[72] Lindh AU, Beckman K, Carlborg A, Waern M, Salander Renberg E, Dahlin M, Predicting suicide: a comparison between clinical suicide risk assessment and the Suicide Intent Scale. J Affect Disord 2020; 263: 445–9. 31969276 10.1016/j.jad.2019.11.131

[73] Lopez-Morinigo JD, Ayesa-Arriola R, Torres-Romano B, Fernandes AC, Shetty H, Broadbent M, Risk assessment and suicide by patients with schizophrenia in secondary mental healthcare: a case-control study. BMJ Open 2016; 6: e011929. 10.1136/bmjopen-2016-011929 PMC5051464 27678536

[74] Lopez-Morinigo JD, Fernandes AC, Shetty H, Ayesa-Arriola R, Bari A, Stewart R, Can risk assessment predict suicide in secondary mental healthcare? Findings from the South London and Maudsley NHS Foundation Trust Biomedical Research Centre (SLaM BRC) Case Register. Soc Psychiatry Psychiatr Epidemiol 2018; 53: 1161–71. 29860569 10.1007/s00127-018-1536-8 PMC6208937

[75] Lukaschek K, Baumert J, Krawitz M, Erazo N, Forstl H, Ladwig KH. Determinants of completed railway suicides by psychiatric in-patients: case-control study. Br J Psychiatry 2014; 205: 398–406. 25257065 10.1192/bjp.bp.113.139352

[76] Madsen T, Agerbo E, Mortensen PB, Nordentoft M. Predictors of psychiatric inpatient suicide: a national prospective register-based study. J Clin Psychiatry 2012; 73: 144–51. 21903026 10.4088/JCP.10m06473

[77] McCarthy JF, Bossarte RM, Katz IR, Thompson C, Kemp J, Hannemann CM, Predictive modeling and concentration of the risk of suicide: implications for preventive interventions in the US department of veterans affairs. Am J Public Health 2015; 105: 1935–42. 26066914 10.2105/AJPH.2015.302737 PMC4539821

[78] McCoy TH J, Castro VM, Roberson AM, Snapper LA, Perlis RH. Improving prediction of suicide and accidental death after discharge from general hospitals with natural language processing. JAMA Psychiatry 2016; 73: 1064–71. 27626235 10.1001/jamapsychiatry.2016.2172 PMC9980717

[79] Modestin J, Kopp W. A study of clinical suicide. J Nerv Ment Dis 1988; 176: 668–74. 3183651

[80] Modestin J, Kopp W. Study on suicide in depressed inpatients. J Affect Disord 1988; 15: 157–62. 2975686 10.1016/0165-0327(88)90084-5

[81] Modestin J, Zarro I, Waldvogel D. A study of suicide in schizophrenic in-patients. Br J Psychiatry 1992; 160: 398–401. 1562868 10.1192/bjp.160.3.398

[82] Motto JA, Heilbron DC, Juster RP. Development of a clinical instrument to estimate suicide risk. Am J Psychiatry 1985; 142: 680–6. 4003585 10.1176/ajp.142.6.680

[83] Motto JA, Bostrom A. Empirical indicators of near-term suicide risk. Crisis 1990; 11: 52–9. 2376147

[84] Neuner T, Schmid R, Wolfersdorf M, Spiessl H. Predicting inpatient suicides and suicide attempts by using clinical routine data? General Hosp Psychiatry 2008; 30: 324–30. 10.1016/j.genhosppsych.2008.03.003 18585535

[85] Nordentoft M, Breum L, Munck LK, Nordestgaard AG, Hunding A, Laursen Bjaeldager PA. High mortality by natural and unnatural causes: a 10 year follow up study of patients admitted to a poisoning treatment centre after suicide attempts. BMJ 1993; 306: 1637–41. 8324430 10.1136/bmj.306.6893.1637 PMC1678054

[86] Nordstrom P, Asberg M, Aberg-Wistedt A, Nordin C. Attempted suicide predicts suicide risk in mood disorders. Acta Psychiatr Scand 1995; 92: 345–50. 8619338 10.1111/j.1600-0447.1995.tb09595.x

[87] Park SJ, Lee HB, Ahn MH, Park S, Choi EJ, Lee HJ, Identifying clinical correlates for suicide among epilepsy patients in South Korea: a case-control study. Epilepsia 2015; 56: 1966–72. 26530473 10.1111/epi.13226

[88] Pokorny AD. Prediction of suicide in psychiatric patients. Report of a prospective study. Arch Gen Psychiatry 1983; 40: 249–57. 6830404 10.1001/archpsyc.1983.01790030019002

[89] Powell J, Geddes J, Deeks J, Goldacre M, Hawton K. Suicide in psychiatric hospital in-patients. Risk factors and their predictive power. Br J Psychiatry 2000; 176: 266–72. 10755075 10.1192/bjp.176.3.266

[90] Pratt D, Appleby L, Piper M, Webb R, Shaw J. Suicide in recently released prisoners: a case-control study. Psychol Med 2010; 40: 827–35. 19719900 10.1017/S0033291709991048

[91] Randall JR, Sareen J, Chateau D, Bolton JM. Predicting future suicide: clinician opinion versus a standardized assessment tool. Suicide Life Threat Behav 2019; 49: 941–51. 29920749 10.1111/sltb.12481

[92] Rosen DH. The serious suicide attempt. Five-year follow-up study of 886 patients. JAMA 1976; 235: 2105–9. 946536 10.1001/jama.235.19.2105

[93] Roy A. Suicide in chronic schizophrenia. Br J Psychiatry 1982; 141: 171–7. 6126237 10.1192/bjp.141.2.171

[94] Sanderson M, Bulloch AGM, Wang J, Williamson T, Patten SB. Predicting death by suicide using administrative health care system data: can feedforward neural network models improve upon logistic regression models? J Affect Disord 2019; 257: 741–7. 31394413 10.1016/j.jad.2019.07.063

[95] Shaffer JW, Perlin S, Schmidt CW Jr, Stephens JH. The prediction of suicide in schizophrenia. J Nerv Ment Dis 1974; 159: 349–55. 4436660 10.1097/00005053-197411000-00005

[96] Sharma V, Persad E, Kueneman K. A closer look at inpatient suicide. J Affect Disord 1998; 47: 123–9. 9476752 10.1016/s0165-0327(97)00131-6

[97] Simon GE, Johnson E, Lawrence JM, Rossom RC, Ahmedani B, Lynch FL, Predicting suicide attempts and suicide deaths following outpatient visits using electronic health records. Am J Psychiatry 2018; 175: 951–60. 29792051 10.1176/appi.ajp.2018.17101167 PMC6167136

[98] Spiessl H, Hubner-Liebermann B, Cording C. Suicidal behaviour of psychiatric in-patients. Acta Psychiatr Scand 2002; 106: 134–8. 12121211 10.1034/j.1600-0447.2002.02270.x

[99] Steblaj A, Tavcar R, Dernovsek MZ. Predictors of suicide in psychiatric hospital. Acta Psychiatr Scand 1999; 100: 383–8. 10563456 10.1111/j.1600-0447.1999.tb10882.x

[100] Steeg S, Kapur N, Webb R, Applegate E, Stewart SL, Hawton K, The development of a population-level clinical screening tool for self-harm repetition and suicide: the ReACT Self-Harm Rule. Psychol Med 2012; 42: 2383–94. 22394511 10.1017/S0033291712000347

[101] Stephens JH, Richard P, McHugh PR. Suicide in patients hospitalized for schizophrenia: 1913–1940. J Nerv Ment Dis 1999; 187: 10–4. 9952248 10.1097/00005053-199901000-00003

[102] Walby FA, Odegaard E, Mehlum L. Psychiatric comorbidity may not predict suicide during and after hospitalization. A nested case-control study with blinded raters. J Affect Disord 2006; 92: 253–60. 16546263 10.1016/j.jad.2006.02.005

[103] Weiser M, Goldberg S, Werbeloff N, Fenchel D, Reichenberg A, Shelef L, Risk of completed suicide in 89,049 young males assessed by a mental health professional. Eur Neuropsychopharmacol 2016; 26: 341–9. 26712325 10.1016/j.euroneuro.2015.12.001

[104] Windfuhr K, While D, Kapur N, Ashcroft DM, Kontopantelis E, Carr MJ, Suicide risk linked with clinical consultation frequency, psychiatric diagnoses and psychotropic medication prescribing in a national study of primary-care patients. Psychol Med 2016; 46: 3407–17. 27650367 10.1017/S0033291716001823

[105] Winkler P, Mlada K, Csemy L, Nechanska B, Hoschl C. Suicides following inpatient psychiatric hospitalization: a nationwide case control study. J Affect Disord 2015; 184: 164–9. 26093829 10.1016/j.jad.2015.05.039

[106] Rosenthal J. Qualitative descriptors of strength of association and effect size. J Soc Serv Res 1996; 21: 37–57.

[107] Cohen J. Statistical approaches to suicidal risk factor analysis. Ann NY Acad Sci 1986; 487: 34–41. 3471164 10.1111/j.1749-6632.1986.tb27883.x

