# Supplementary material for: Meta-analysis of the strength of exploratory suicide prediction models; from clinicians to computers
Source: BJPsych Open. 2021 Jan 7;7(1):e26. doi: 10.1192/bjo.2020.162 (PMC8058929; doi:10.1192/bjo.2020.162)
Supplement: Supplementary file 1 [file S2056472420001623sup001.zip › Suppelmentary Table 1. Table of included studies reporting exploratory suicide prediction models.docx]

| Table 1. Table of included studies reporting exploratory suicide prediction models | | | | | | | | | |
| --- | --- | --- | --- | --- | --- | --- | --- | --- | --- |
| Study N. Author (year of publication) | Location | Method | Patient group | Subjects  n. | Suicides  n. | Initial risk factors N. | Method of suicide prediction model | Risk factors in the suicide prediction model | Odds Ratio |
| 1. Ahmedani et al. (2017)^20^ | Research network, USA | case control | primary care | 270074 | 2674 | 24 | logistic regression & experimental scale | two or more major 19 physical health conditions | 3.4 (3.1-3.6) |
| 2. Allgulander & Fisher (1990) ^21^ | Stockholm County, Sweden | cohort | men previously admitted for intentional self-poisoning with psychoactive drugs | 3381 | 231 | 23 | upper quintile of cox proportional hazards regression | older age  prior suicide attempt  prescription drug abuse | 2.2 (1.6-2.9) |
| 2. Allgulander & Fisher (1990) ^21^ | Stockholm County, Sweden | cohort | women previously admitted for intentional self-poisoning with psychoactive drugs | 5514 | 262 | 23 | upper quintile of cox proportional hazards regression | older age  prior suicide attempt  neurotic/personality disorder  affective disorder  drug + alcohol at time of poisoning  >1 month index admission | 3.0 (2.3-3.8) |
| 3. Appleby et al. (1999)^22^ | Manchester, United Kingdom | matched case control | previously admitted psychiatric patients | 298 | 149 |  | clinical judgment |  | 2.0 (.8-4.8) |
| 4. Bickley et al (2013)^23^ | England, United Kingdom | case control | previously admitted psychiatric patients | 200 | 100 | 90 | logistic regression & experimental scale | age ≥40  male  self-harm  secondary diagnosis  recent adverse life events  short admission  non-enhanced care | 12.5 (5.4-29.1) |
| 5. Blaauw et al. (2005) ^24^ | Netherlands | case control | prisoners | 281 | 48 | 27 | logistic regression | history of psychiatric care  age >40  violent offence  homelessness  more than one incarceration  hard drug abuse | 19.7 (8.9-43.8) |
| 6. Bruer et al (2018) ^25^ | Ontario, Canada | case control | current psychiatric inpatients | 294 | 98 | 38 | logistic regression | male sex  non-hazardous drinking  severe self-harm  self-harm  mood diagnosis  loss of interest  recent admission to two hospitals  discrepancy in history of hospital care | 10.8 (6.0-19.4) |
| 7. Buglass & Horton (1974)^26^ | Edinburgh, United Kingdom | cohort | previously admitted to a poisoning centre | 766 | 7 | 24 | experimental scale | sociopathy  problematic alcohol use  previous inpatient psychiatric treatment  previous outpatient psychiatric treatment  parasuicide resulting in admission  not living with a relative | 3.8 (.5-32.0) |
| 8. Buglass & McCulloch (1970)^27^ | Edinburgh,  United Kingdom | cohort | men previously admitted to a poisoning centre | 175 | 10 | 34 | experimental scale | violence in key relationship  alcoholism  alcohol at time of suicide attempt | 5.1 (1.3-19.8) |
| 8. Buglass & McCulloch (1970)^27^ | Edinburgh, United Kingdom | cohort | women  previously admitted to a poisoning centre | 336 | 7 | 34 | experimental scale | previous attempted suicide previous psychiatric treatment  psychopathy  drug addiction  unstable accommodation  childhood separation from mother  childhood separation from father  poor work record  violence in key relationship | 3.0 (.6-15.9) |
| 9. Chang et al. (2020) ^28^ | Taiwan | matched case control | national sample | 278150 | 55630 | 25 | logistic regression & experimental scale | physical illness  psychiatric illness | 6.7 (6.5-6.9) |
| 10. Choi et al (2018)^29^ | South Korea | cohort | national health insurance sample | 819951 | 2546 | 39 | cox proportional hazards regression | male sex  older age  medical aid insurance  low household income  disability  eight physical illness codes | 6.8 (5.3-8.6) |
| 10. Choi et al (2018)^29^ | South Korea | cohort | national health insurance sample | 819951 | 2546 | 39 | machine learning | not reported | 3.1 (3.0-3.2) |
| 11. Conlon et al. (2007) ^30^ | Galway, Ireland | matched case control | adults with mental illness | 78 | 39 |  | clinical judgment |  | 6.1 (1.8-20.5) |
| 12. Dean et al. (1967)^31^ | Denver, USA | matched case control | current psychiatric inpatients | 132 | 16 | 48 | experimental scale | males aged 40-60  psychosis  under care  admitted  involuntary status  times admitted  married  years of education  protestant religion  higher income  unstable employment  preoccupation  slowing  appropriate language  anger  depression  apathy  friendly attitude  appropriate behavior  not against  not hostile  more effectiveness  more cooperative  lack of denial  external stress  danger to self | 21.9 (6.2-77.2) |
| 13. De Hert et al. (2001)^32^ | Kortenberg, Belgium | matched case control | Previously admitted patients with schizophrenia | 126 | 63 | 35 | experimental scale | depression  psychotic symptoms | 35.7 (4.6-274.5) |
| 14. DelPozo-Banos et al. (2018)^33^ | Wales, United Kingdom | matched case control | primary health care | 54684 | 2604 | 15 | machine learning | psychotrophic prescription  depression and anxiety  self-harm  hospital admissions  drug and alcohol misuse | 8.2 (7.5-8.9) |
| 15. Didham et al. (2006)  ^34^ | Dunedin, New Zealand | matched nested case control | primary health care | 884 | 221 | 49 | logistic regression | previous psychiatric admission  depression, suicidal ideation or self-harm  admission for self-harm  sedatives | 11.4 (8.5-15.2) |
| 15. Didham et al. (2006)  ^34^ | Dunedin, New Zealand | matched nested case control | primary health care | 884 | 221 | 49 | experimental scale | depression  suicidal ideation  self-harm | 15.0 (5.7-39.2) |
| 16. Dong et al. (2005)^35^ | Hong Kong, China | matched case control | current psychiatric inpatients | 184 | 92 | 78 | logistic regression & experimental scale | deliberate self-harm  admitted for suicidal behavior  depressive symptoms  absent without leave  extrapyramidal side effects | 94.8 (5.7 - 1577) |
| 17. Drake et al (1984)^36^ | Massachusetts, USA | case control | previously admitted patients with schizophrenia | 104 | 15 | 44 | logistic regression | suicide threats  hopelessness  fears of mental disintegration | 32.2 (8.1 - 128) |
| 18. Dutta et al. (2011)^37^ | multiple locations, United Kingdom | cohort | patients with first-episode psychosis | 1575 | 33 | 74 | cox proportional hazards regression & experimental scale | poor premorbid adjustment  difficult rapport  persecutory delusions  grandiose delusions  delusions of passivity  any auditory hallucinations  reckless activity  dysphoria  suicidal ideation  family history of psychosis | 9.1 (3.0-28.0) |
| 19. Epstein et al. (1973)^38^ | John Hopkins University, USA | matched case control | medical students | 33 | 9 |  | clinical judgment |  | 297 (11-7975) |
| 20. Farberow et al. (1966)^39^ | Veterans Affairs Neuropsychiatric hospitals, USA | case control | male neuropsychiatric inpatients | 438 | 218 |  | clinical judgment |  | 15.2 (9.4-24.7) |
| 21. Farberow & MacKinnon (1974)^40^ | Veterans Affairs Neuropsychiatric hospitals, USA | case control | previously admitted psychiatric patients | 187 | 93 | 81 | experimental scale | younger age  drug or alcohol issues  early return from pass  absent without leave  recent divorce  suicidal history  nervousness or anxiety  occupational problems  recent illness  death or a loved one  reluctant to be discharged | 138.9 (8.4-2306) |
| 22. Fazel et al (2019)^41^ | Sweden | cohort | adults with serious mental illness | 58 771 | 494 | 34 | logistic regression | male sex  younger age  violent crime  drug use  alcohol use  self-harm  more education  parental substance use  parental suicide  recent antipsychotic  recent antidepressant  current inpatient  length of first inpatient stay >7 days  number of episodes  benefit receipt  parental psychiatric hospitalization  comorbid depression | 4.4 (3.7-5.3) |
| 23. Fosse et al. (2017)^42^ | Blakstad, Noway | matched case control | previously admitted psychiatric patients | 160 | 40 | 52 | logistic regression | brief suicide checklist  admitted with suicidal problems  schizophrenia or depression | 33.7 (12.5-90.50 |
| 24. Funahashi et al. (2000)^43^ | Nagoya, Japan | matched case control | current and previously admitted patients with schizophrenia | 143 | 74 | 47 | logistic regression | birth order (being a middle child)  suicidal ideation  anxiety | 103 (30.8-346) |
| 25. Goldberger et al. (2015)^44^ | Israel | cohort | current and previously admitted psychiatric inpatients | 158800 | 2650 | 26 | logistic regression | male  immigrant group  drug and alcohol addiction  non-affective psychosis  affective disorder  other non-organic disorders  older age  more hospitalizations  suicide attempt  shorter follow up | 8.6 (8.4-8.8) |
| 26. Goldstein et al. (1991) ^45^ | Iowa City, USA | cohort | previously admitted affective disorder | 1906 | 46 | 21 | logistic regression | male  suicide ideation  not bipolar disorder  unfavorable discharge  unipolar depression with family  history of mania  number of prior suicide attempts | 10.3 (1.1-94.1) |
| 27. Gradus et al. (2019) ^46^ | Denmark | matched case control | men in the general population | 140743 | 10152 | 2 554 | machine learning | antidepressants  hypnotics  drug use  antipsychotics  older than 14  Poisoning at 24 month  second income quartile  anxiolytics  suicide attempt at 24 months  severe reaction to stress or adjustment disorder  alcohol use | 15.8 (14.9-16.6) |
| 27. Gradus et al. (2019) ^46^ | Denmark | matched case control | women in the general population | 138543 | 3951 | 2 554 | machine learning | antidepressants  hypnotics  antipsychotics  anxiolytics  poisoning in the last 48 months  schizophrenia  drug use  age over 50  major depression  antibacterial medication  suicide attempt  opiods at 6 months  age under 40  alcohol related disorder | 30.7(28.7-32.9) |
| 28. Harriss & Hawton (2005)^47^ | Oxford, United Kingdom | cohort | men with deliberate self-harm | 1049 | 29 | 27 | logistic regression | suicide intent scale  alcohol misuse | 4.9 (1.2-20.5) |
| 28. Harriss & Hawton (2005)^47^ | Oxford, United Kingdom | cohort | women with deliberate self-harm | 1440 | 24 | 27 | logistic regression | suicide intent scale  past psychiatric treatment  older age | 12.2 (4.2-36.0) |
| 29. Haste et al. (1998) ^48^ | Research network, United Kingdom | matched case control | general practices patients | 1347 | 339 | 45 | logistic regression & experimental scale | serious mental illness  no treatment for serious mental illness | 20.8 (3.2-136.1) |
| 30. Hawton et al. (1993)^49^ | Ebinburgh, United Kingdom | matched case control | young people with deliberate self-harm | 186 | 62 | 28 | logistic regression & experimental scale | previous inpatient  psychiatric treatment  substance misuse | 5.0 (2.3-10.9) |
| 31. Holmstrand et al. (2015)^50^ | Lund, Sweden | cohort | men in the general population | 3563 | 68 | 6 | logistic regression | alcohol use  depression | 25.1 (11.3-55.7) |
| 31. Holmstrand et al. (2015)^50^ | Lund, Sweden | cohort | general population | 3563 | 68 | 7 | experimental scale | organic brain disorder  psychosis  depression  anxiety  alcohol use  other psychiatric disorder | 4.6 (2.8-7.7) |
| 32. Humber et al. (2013)^51^ | England and Wales, United Kingdom | matched case control | prisoners | 440 | 220 | 25 | logistic regression & experimental scale | history of self-harm  occupying a single cell  remand status  non-white ethnicity  previous mental health service contact | 14.6 (3.4-62.4) |
| 32. Humber et al. (2013)^51^ | England and Wales, United Kingdom | matched case control | prisoners | 440 | 220 |  | clinical judgment |  | 7.1 (4.4-11.2) |
| 33. Hung et al. (2015)^52^ | Taipei, Taiwan | cohort | older people in primary care | 101857 | 93 | 18 | cox proportional hazards regression | male  less education  income subsidy  depressed mood  insomnia | 4.7 (3.1-7.2) |
| 34. Hunt et al (2007) ^53^ | England, United Kingdom | case control | current psychiatric inpatients | 444 | 222 | 90 | logistic regression & experimental scale | male sex  affective disorder  deliberate self-harm  employed | 4.6 (3.1 – 6.8) |
| 35. Hunt et al (2009) ^54^ | England, United Kingdom | case control | previously admitted psychiatric patients | 476 | 238 | 90 | logistic regression & experimental scale | male sex  self-harm  affective disorder  psychiatric comorbidity  recent psychiatric care  suicidal ideation  self discharged  missed appointment | 3.8 (2.4 – 6.0) |
| 36. Hunt et al (2013) ^55^ | England, United Kingdom | case control | recently admitted  psychiatric patients | 214 | 107 | 90 | logistic regression & experimental scale | male  self-harm  recent life events  short illness (< 12 months) | 5.8 (3.2-10.6) |
| 37. Ilgen et al (2009)^56^ | Veterans, USA | cohort | veterans diagnosed with depression | 589825 | 1 275 | 8 | Bayesian data mining and decision tree | male  substance use  non-African American  inpatient stay last 12 months | 3.8 (3.0-4.7) |
| 38. Kan et al 2007^57^ | Hong Kong, China | matched case control | previously admitted psychiatric patients | 194 | 97 | 35 | logistic regression & experimental scale | admitted for deliberate self-harm/suicide idea  living alone  occupational stress  involuntary admission  previous deliberate self-harm  lost to follow-up | 6.5 (3.1-13.7) |
| 39. Kauppila et al (2020)^58^ | Nordic countries | cohort | people who have had bariatric surgery | 49977 | 98 | 11 | experimental scale | depression or anxiety  mania, bipolar, psychosis or schizophrenia  personality disorder  substance use | 10.3 (6.1-17.4) |
| 40. Kessler et al. (2015)^59^ | Multiple military hospitals, USA | cohort | veterans who previously admitted psychiatric patients | 53769 | 68 | 421 | machine learning | male  older enlistment  higher military enlistment score  number of registered pistols  verbal assault offences  non-violent gun charges  prior suicide attempt  visits with suicide ideation  longer outpatient treatment  antidepressant treatment  number of prior psychiatric hospitalization  recent hospitalization in <12 months  major depression  somatoform or dissociative disorder  non-military psychiatric hospitalization  current non-PTSD diagnosis  current suicidal ideation  current non-affective psychosis  current somatoform or dissociative disorder  current hearing loss | 21.6 (13.4-34.9) |
| 41. Kessler et al. (2017)^60^ | Veterans, USA | case cohort | veterans | 2102137 | 6359 | 381 | machine learning | 61 clinical variables | 30.1 (25.7-35.3) |
| 42. Kessler et al. (2017)^61^ | Veterans, USA | case control | veterans who were previously admitted psychiatric patients or having psychiatric outpatient care | 316686 | 234 | 1000 | machine learning | suicidality  depression  non-affective psychosis  bipolar disorder  visit to a psychiatrist  ill defined disorder  any physical disorder  non-affective psychosis  personality disorder  anticonvulsants  anxiety  alcohol or narcotic treatment  multiple crime perpetration | 4.8 (3.7-6.2) |
| 43. Kessler et al. (2020)^62^ | Veterans, USA | case control | veterans who previously admitted psychiatric patients | 195349 | 771 | 8071 | machine learning | over 50 including  various measures of suicide attempt  high risk flag  psychiatric hospitalization  various measures of drug dependency  neurotic personality  other non-psychotic disorders  schizophrenia  current mood disorder  non-Hispanic black  non-Hispanic white  housing problems  psychological or physical stress  various measures county social capital  county suicide rate  pain diagnosis  heart disease  chronic obstructive airway disease | 5.5 (4.6-6.5) |
| 44. Kim et al. (2012)^63^ | Veterans, USA | matched case control | veterans with depression | 636 | 324 | 69 | logistic regression | not Black  not Hispanic  older  anxiety disorder  no PTSD  not service connected  alcohol  psychiatric hospitalization  non psychiatric hospitalization  more non mental health visits  psychotropic medication  psychotic symptoms  no chronic or acute pain  alcohol abuse  prescription drug misuse  family problems  death of a family member  poor access to health care  occupational problems  suicide attempt  suicide ideation  homicidal thoughts  mental health hospitalization considered  no veterans substance abuse treatment  Attends AA or similar | 9.5 (6.8 – 13.2) |
| 45. King et al. (2001)^64^ | Wessex, United Kingdom | matched case control | previously admitted psychiatric patients | 665 | 234 | 105 | logistic regression & experimental scale | loss of key worker  becoming unemployed  new relationship problems  history of DSH  not planned discharge  DSH during admission  not white  suicidal ideas at index admission  living alone  hopelessness  admitted under a new consultant | 4.7 (3.1-7.3) |
| 46. King et al (2001)a^65^ | Wessex, United Kingdom | matched case control | current  psychiatric in patients | 165 | 59 | 60 | logistic regression & experimental scale | deliberate self-harm before admission  involuntary admission  police involvement in admission  depressive symptoms  loss of key worker  absent without leave  violence to property | 11.9 (1.4 - 101) |
| 47. Kjelsberg et al. (1991)^66^ | Oslo, Norway | matched case control | previously admitted with borderline personality disorder | 42 | 21 | 71 | experimental scale | earlier serious suicide attempt  lack of therapist contact  unplanned discharge  childhood loss  longer hospitalization | 129 (6.6-2503) |
| 48. Kjelsberg et al. (1994)^67^ | Oslo, Norway | matched case control | previously admitted adolescents | 70 | 35 | 125 | logistic regression & experimental scale | lack of parental support  parental verbal abuse  serious loss before 2 years of age  depressive symptoms  help-rejecting  learning difficulties  poor self-esteem  immature defenses | 36.0 (8.7-149) |
| 49. Krupinski et al. (1998)^68^ | Munich, Germany | cohort | current inpatients with depressive psychosis | 3282 | 30 | 272 | discriminant function analysis | suicidal tendency  previous suicide attempt  no early waking  no retarded thinking  no recent inpatient treatment  older age  no constipation  more siblings  no children  married  number of inpatient treatments  female  siblings  current stress  drug or alcohol  shorter illness | 21.8 (7.6 – 62.5) |
| 50. Krupinski et al. (2000)^69^ | Munich, Germany | cohort | current inpatients with schizophrenia | 4621 | 17 | 272 | discriminant function analysis | feelings of numbness  thought insertion  free floating anxiety  depressed mood  observed depression  suicidality  no delusions  previous suicide attempts  no aggression | 25.5 (8.9-72.8) |
| 51. Levi et al. (2016)^70^ | Israel | case control | current psychiatric inpatients | 1630 | 326 | 14 | logistic regression | schizoaffective disorder  schizophrenia  affective disorder  previous suicide attempt  older age at onset  male sex  previous hospitalisations  involuntary status | 7.7 (5.4-10.9) |
| 52. Lin et al. (2014)^71^ | Taipei, Taiwan | matched case control | current psychiatric inpatients | 203 | 41 | 95 | logistic regression & experimental scale | depressed mood  psychomotor retardation  loss of energy  psychotic symptoms  family history of psychosis | 2.3 (1.0 -5.0) |
| 53. Lindh et al (2020)^72^ | Stockholm, Sweden | cohort | people with deliberate self-harm | 422 | 14 |  | clinical judgment |  | 4.1 (1.3-13.4) |
| 54. Lopez-Morinigo et al. (2016)  ^73^ | London, United Kingdom | case control | patients with schizophrenia | 245 | 31 | 37 | logistic regression | younger age at presentation  suicidal history  suicide ideation | 2.3 (0.2-23.3) |
| 55. Lopez-Morinigo et al. (2018)^74^ | London, United Kingdom | cohort | secondary mental health care | 13759 | 81 | 32 | cox proportional hazards regression & experimental scale | male sex  suicidal history  violent method  plan to end life  suicidal ideation  hopelessness  distress  no control over life  impulsivity  significant loss  disengagement  recent discharge | 3.2 (2.0-5.1) |
| 56. Lukaschek et al. (2014)^75^ | Psychiatric hospitals, Germany | matched case control | current psychiatric inpatients | 202 | 101 | 54 | logistic regression | unemployment  change of therapist  poor response to treatment  suicide ideas  polypharmacological treatment | 26.4 (13.4-52.1) |
| 57. Madsen  et al. (2011) ^76^ | Denmark | cohort | current psychiatric inpatients | 126382 | 279 | 43 | cox proportional hazards regression & experimental scale | male sex  older age  employed  more education  married  affective disorder  secondary diagnosis of personality disorder  previous suicide attempt  recent suicide attempt  private psychologist | 1.8 (1.2-2.6) |
| 58. McCarthy et al. (2105)^77^ | Veterans, USA | case control | veterans | 1059184 | 3180 | 381 | logistic regression | younger  divorced  urban  depression  schizophrenia  bipolar disorder  post-traumatic stress disorder  personality disorder  substance use disorder  other anxiety disorder  other psychiatric disorder  pain  sleep problem  traumatic brain injury | 45.9 (37.9 -55.5) |
| 59. McCoy et al. (2016)^78^ | Boston, USA | cohort | people discharged from general hospitals | 458053 | 235 | 11 | cox proportional hazards regression & experimental scale | male  white  older age  comorbidity  admitted for psychiatric diagnosis  recent psychiatric visits  any psychiatric visit  any outpatient visits in last 12m  emergency department visits in last 12m  lack of positive valence natural language  negative valence natural language | 5.1 (5.0-5.1) |
| 60. Modestin & Kopp (1988)^79^ | Bern, Switzerland | case control | current psychiatric inpatients | 298 | 149 | 34 | logistic regression | suicidal behavior in index admission  male  diagnosis  number of suicide attempts  duration of index hospitalization  social loss  family history of suicide | 18.8 (10.5-33.6) |
| 61. Modestin & Kopp (1988)^80^ | Bern, Switzerland | case control | current psychiatric inpatients with depression | 125 | 75 | 35 | logistic regression | suicidal behavior during index admission  male sex  number of hospitalisations  suicide attempt at index admission  broken home  social exits | 9.4 (4.1-21.7) |
| 62. Modestin et al. (1992)^81^ | Bern, Switzerland | matched case control | current psychiatric inpatients with schizophrenia | 106 | 53 | 41 | logistic regression | suicidal behavior in index admission  early vocational difficulties  duration of index admission | 26.7 (8.9-80.7) |
| 63. Motto, et al (1985) ^82^ | San Francisco, USA | cohort | previously admitted depressive or suicidal patients | 2753 | 136 | 162 | logistic regression & experimental scale | older age  skilled worker  more wealthy  family psychiatric history  non-heterosexual  previous psychiatric admissions  failed prior psychological help  financial strain  special stress  hypersomnia  weight change  persecutory or referential ideas  suicidal impulses  seriousness of suicide attempt  negative countertransference | 8.4 (3.9 – 18.0) |
| 64. Motto & Bostrom (1990)^83^ | San Francisco,  USA | cohort | previously admitted depressive or suicidal patients | 2999 | 38 | 104 | logistic regression & experimental scale | prior psychiatric hospitalization  considering lethal method  suicidal ideas  divorced  financial stress  feels a burden  negative countertransference  severe crying or unable to cry  persecutory or referential ideas | 16.5 (6.0-45.4) |
| 65. Neuner et al. (2008) ^84^ | Regensburg, Germany | cohort | current psychiatric inpatients | 20543 | 41 | 74 | logistic regression | treatment resistance  previous suicide attempt  severe side effects  previous supportive psychotherapy | 3.4 (.2-55.3) |
| 66. Nordentoft et al (1993)^85^. | Bispebjerg, Denmark | cohort | previously admitted to a poisoning centre | 974 | 103 | 21 | cox proportional hazards regression & experimental scale | living alone  manic depressive illness  ≥ 2 previous suicide attempts | 2.4 (1.6-3.6) |
| 67. Nordstrom et al. (1995)^86^ | Stockholm, Sweden | cohort | previously admitted for mood disorder | 346 | 27 | 12 | survival analysis & experimental scale | major affective disorder  melancholia  suicide attempt | 3.4 (1.2-9.9) |
| 68. Park et al. (2015) ^87^ | Seoul, Korea | matched case control | patients with epilepsy | 296 | 74 | 32 | logistic regression | frequent of seizures  lack of aura prior to seizure  temporal lobe epilepsy  levetiracetam  antidepressants | 13.9 (8.3-23.2) |
| 69. Pokorny (1983)^88^ | Houston, USA | cohort | previously admitted psychiatric patients who were veterans | 4800 | 67 | 153 | discriminant function analysis | prior suicide attempt  affective disorder or schizophrenia  suicide list  not married | 3.6 (2.2-5.9) |
| 69. Pokorny (1983)^88^ | Houston, USA | cohort | previously admitted veteran psychiatric patients who were veterans | 4800 | 67 | 153 | experimental scale | Non black  diagnosis of affective disorder or schizophrenia  alcoholism or drug abuse  Male  Unmarried | 4.6 (2.6-8.2) |
| 70. Powell et al. (2000)^89^ | Oxford, United Kingdom | case control | current psychiatric inpatients | 224 | 112 | 74 | logistic regression | suicidal thoughts  suicide plan  recent self-harm  recent bereavement  delusions  chronic mental illness  family history of suicide | 32.6 (4.3-246) |
| 71. Pratt et al. (2010) ^90^ | England and Wales, United Kingdom | matched case control | released prisoners | 208 | 104 |  | clinical judgment |  | 2.7 (1.3-5.7) |
| 72. Randall et al (2018)  ^91^ | Winnipeg, Canada | cohort | people presenting to hospitals with mental health needs | 2643 | 20 | 9 | logistic regression & experimental scale | suicide attempt  imminent suicide plans  suicide ideation | 2.9 (1.1-7.5) |
| 72. Randall et al (2018)  ^91^ | Winnipeg, Canada | cohort | people presenting to hospitals with mental health needs | 2643 | 20 |  | clinical judgment |  | 1.05 (.9-1.3) |
| 73. Rosen (1976) ^92^ | Edinburgh, United Kingdom | cohort | discharged from a poisoning centre | 886 | 34 | 10 | experimental scale | medically serious suicide attempt  psychiatrically serious suicide attempt | 2.1 (1.0 – 4.4) |
| 74. Roy (1982)^93^ | Toronto,  Canada | matched case control | discharged psychiatric patients | 146 | 86 | 16 | experimental scale | schizophrenic symptoms  suicidal  depressive episode | 3.0 (.9 – 9.4) |
| 74. Roy (1982)^93^ | Toronto,  Canada | matched case control | discharged patients with schizophrenia | 57 | 29 | 16 | experimental scale | schizophrenic symptoms  suicidal  depressive episode | 3.7 (1.2 – 11.4) |
| 75. Sanderson et al. (2019)^94^ | Alberta, Canada | case control | primary health care | 39028 | 3548 | 101 | logistic regression | not reported | 9.0 (8.3-9.7) |
| 75. Sanderson et al. (2019)^94^ | Alberta, Canada | case control | primary health care | 39028 | 3548 | 101 | machine learning | not reported | 9.9 (9.2-10.7) |
| 76. Shaffer et al. (1974) ^95^ | Baltimore,  USA | case control | discharged patients with schizophrenia | 87 | 12 | 32 | linear regression | previous suicide attempts  alcohol problems  male sex  older age | 35.0 (11.8-104) |
| 76. Shaffer et al. (1974) ^95^ | Baltimore,  USA | case control | discharged patients with schizophrenia | 87 | 12 |  | clinical judgment |  | 4.9 (2.1-11.4) |
| 77. Sharma et al. (1998)^96^ | Ontario, Canada | matched case control | current psychiatric inpatients | 88 | 44 |  | clinical judgment |  | 3.4 (1.4-8.2) |
| 78. Simon et al. (2018)^97^ | Health insurers, USA | cohort | secondary mental health care | 6679128 | 1529 | 313 | machine learning | 43 risk factors including:  Suicide attempt diagnosis in past year  Benzodiazepine prescription in past 3 months  Mental health emergency department visit in past 3 months  Second-generation antipsychotic prescription in past 5 years  Mental health inpatient stay in past 5 year  Mental health inpatient stay in past 3 mon ths  Mental health inpatient stay in past year  Alcohol use disorder diagnosis in past 5 years  Antidepressant prescription in past 3 months  PHQ-9 item 9 score=3 with PHQ-8 score  PHQ-9 item 9 score=1 with age  Depression diagnosis in past 5 years with  Suicide attempt diagnosis in past 5 years with Charlson score  PHQ-9 item 9 score=2 with age  Anxiety disorder diagnosis in past 5 years with age | 29.7 (26.4 – 33.5) |
| 78. Simon et al. (2018) ^97^ | Health insurers, USA | cohort | primary mental health care | 6297465 | 856 | 313 | machine learning | 29 risk factors including:  Mental health emergency department visit in past 3 months  Alcohol abuse diagnosis in past 5 years  Benzodiazepine prescription in past 3 months  Depression diagnosis in past 5 years  Mental health inpatient stay in past year  Injury/poisoning diagnosis in past year  Anxiety disorder diagnosis in past 5 years  PHQ-9 item 9 score=1 with PHQ-8 score  PHQ-9 item 9 score=3 with age  Suicide attempt diagnosis in past 5 years with age  Mental health emergency department visit in past year  PHQ-9 item 9 score=2 with age  PHQ-9 item 9 score=3 with PHQ-8 score  Bipolar disorder diagnosis in past 5 years with age  Depression diagnosis in past 5 years with age | 26.2 (22.2 – 30.9) |
| 79. Spiessl et al. (2002)^98^ | Regensburg, Germany | cohort | current psychiatric inpatients | 21062 | 30 | 70 | logistic regression | schizophrenia  cumulative length of stay  previous suicide attempt  part time employment  training/retraining | 5.2 (.3-86.6) |
| 80. Steblaj et al. (1999)^99^ | Ljubljana, Slovenia | matched case control | current psychiatric inpatients with schizophrenia | 72 | 36 | 38 | logistic regression | loss of insight  depression  previous suicidal behavior  unsatisfactory family relationships | 17.3 (4.4-67.3) |
| 80. Steblaj et al. (1999)^99^ | Ljubljana, Slovenia | matched case control | current psychiatric inpatients with affective psychoses | 46 | 23 | 38 | logistic regression | loss of insight  depression | 44.4 (8.0-247) |
| 81. Steeg et al. (2012)^100^ | Manchester, Oxford and Derby, UK | cohort | presented to hospital after deliberate self-harm | 18680 | 92 | 31 | logistic regression & experimental scale | recent self-harm (<1year)  living alone/ homeless  cutting as a method of harm  treatment for a current psychiatric disorder | 1.8 (.8-4.1) |
| 82. Stephens, et al. (1999)^101^ | Baltimore, USA | cohort | admitted with schizophrenia | 1212 | 28 | 44 | logistic regression and experimental scale | poor premorbid adjustment  suicidal thoughts  previous suicide attempts  family history of affective illness  depression in hospital  sexual anxieties  psychomotor agitation | 24.2 (10.8 – 54.1) |
| 83. Walby et al (2006) ^102^ | Oslo, Norway | matched case control | current and discharged psychiatric patients | 302 | 136 | 12 | experimental scale | affective disorder  substance use | 2.0 (.9-4.5) |
| 83. Walby et al (2006) ^102^ | Oslo, Norway | matched case control | current and discharged psychiatric patients | 302 | 136 | 12 | logistic regression | depressive disorders  bipolar disorders  divorced or separated  involuntary admitted  significant symptoms < 1year  previous suicide attempt  personality disorder | 2.9 (1.8 – 4.7) |
| 84. Weiser et al (2016) ^103^ | Israel | cohort | young men assessed by mental health professionals | 89049 | 54 | 27 | survival analysis & experimental scale | psychiatric diagnosis  suicide attempts | 8.0 (1.9 – 32.9) |
| 85. Windfuhr et al. (2016) ^104^ | United Kingdom | matched case control | primary care | 49283 | 2384 | 24 | logistic regression and experimental scale | schizophrenia  bipolar disorder  personality disorder  eating disorder  depression  anxiety disorder | 15.2 (9.6-24.1) |
| 86. Winkler et al. (2015) ^105^ | Chech Republic | cohort | psychiatric inpatients andrecently discharged patients | 137290 | 402 | 9 | logistic regression | age 60-69 or 40-49  male  married or divorced  employed  more hospitalizations  anxiety, affective or personality disorders | 2.3 (2.2-2.3) |
